# Oral Health Status among Adult Employees in Kuwait

**DOI:** 10.3290/j.ohpd.b1248897

**Published:** 2021-04-22

**Authors:** Huda Nazar, Maddi Shyama, Jitendra Ariga, Aishah Alsumait

**Affiliations:** a Head, Research and Survey Division, Dental Administration, Ministry of Health, Kuwait. Study design, planning, and conduction, supervision and administration, data collection, analysis and interpretation, wrote and revised manuscript.; b Pedodontist, Research and Survey Division, Dental Administration, Ministry of Health, Kuwait. Data analysis and interpretation, wrote and revised manuscript.; c Director, School Oral Health Program, Kuwait-Forsyth, Kuwait. Wrote and revised manuscript.; d Head, Jaber Al Ahmed School Oral Health Program, Ministry of Health, Kuwait. Revised manuscript.

**Keywords:** adult employees, dental caries, Kuwait, oral health, oral hygiene, oral pain

## Abstract

**Purpose:**

To determine the oral health status among adult employees in Kuwait.

**Materials and Methods:**

This cross-sectional study was performed on a convenience sample (n = 1294) of adult employees. Their ages ranged from 19 to 77 years (mean 36.2). Six trained and calibrated dentists examined them using a mouth mirror and a WHO ball-tip probe. Caries was scored using WHO diagnostic criteria. The debris index simplified (DI-S) score was used to assess oral hygiene status.

**Results:**

Overall, the mean DMFT in the adults was 10.3. The DMFT increased from 7.8 for the age group 19–24 years to 10.7 at 35–44 years and 18.9 at 65–77 years (p < 0.001). Females had slightly higher caries experience (DMFT) (11.0) than did males (10.1) (p = 0.021), and Kuwaitis (11.1) more than non-Kuwaitis (8.9) (p < 0.001). The proportion of caries-free adults was 28%. In multivariate analysis, adults with poor oral hygiene (OR=1.5; 95% CI=1.2-2.1), those with an intermediate-school (grades 6 to 9) or lower level of education (OR=2.6; 95% CI=1.4-4.7), Kuwaitis (OR=1.3; 95% CI=1.0-1.7), those with oral pain (OR=1.4; 95% CI=1.0-1.8), and those needing urgent dental care (OR=4.6; 95% CI=2.6-8.0) were statistically significantly associated with caries risk. About 19.6% of these adults had good, 36.1% fair and 44.4% had poor oral hygiene. Nearly one-third (32.9%) of adults had perceived oral pain at the time of examination.

**Conclusion:**

Implementing oral health programs is needed and efforts should be made to promote oral hygiene practices in workplaces among adults in Kuwait.

Oral health is important for achieving and maintaining good general health and well-being in adults. Poor oral health has a profound effect on overall health and quality of life, and often results in pain, infection, discomfort, and can lead to tooth loss.^12^ According to the WHO, the main risk factors for oral diseases include an unhealthy diet, poor oral hygiene, tobacco use, excess alcohol consumption and social determinants.^35^

Caries is considered the most important universal oral health burden among adults and is listed among the top 100 global burden diseases.^19^ Untreated caries in permanent teeth was a predominant condition worldwide in 2010.^17^ Globally, the prevalence of caries among adults is generally high, as it affects a large proportion of the population in a majority of countries.^27^ The adoption of healthy lifestyle habits, improving living conditions and self-care practices, effective use of fluorides, and establishment of preventive oral health care programs have improved the oral health status among adults in industrialised countries^.22,36^

Oral health in adults is related to their socioeconomic status, income level, urbanisation and access to health services.^20,27^ Adults suffer from the accumulation of untreated oral diseases in developing countries, and decayed teeth (DT) or missing teeth (MT) comprised most of the high caries experience. In contrast, in industrialized countries, filled teeth (FT) comprised most of the measured DMFT index.^34^ Data on the oral health of the adults for the Eastern Mediterranean region (EMR) countries is limited. Nationally representative oral health surveys among the adult population are deficient in the Middle-East countries.

In Kuwait, the dentist to population ratio is at present approximately 1:2370 individuals in the government sector, and in the government and private sector, 1:1577.^21^ Government dental care services for Kuwaiti adults are mainly free of charge. Oral health care for adults is mostly treatment-oriented and no specific preventive procedures or recall systems for regular checkups have been established.^5^ The dental visits of adults have been reported to mainly consist of emergency treatment and for relief of pain.^1,4^

There is a lack of information on the oral health of adults in Kuwait, and there is no national data for adults in Kuwait; it is also limited regarding the dental treatment needs of adults in Kuwait. Information on the oral health status of adults in Kuwait is from an oral health survey that was part of a large national household health survey, the Kuwait Health Survey (N = 26,530), conducted before the Gulf war in 1985.^4^ Caries experience was high among the older age group and the mean caries indices increased with age. The unmet treatment need in adults was substantial.^4^

Since data are lacking on the oral health status of adults in Kuwait, obtaining primary information is important to determine their treatment needs. This in turn is a prerequisite for the appropriate allocation of dental services, planning oral health services and establishing preventive measures. Obtaining baseline information will aid in the formulation of strategies to meet the oral health needs of adults, to provide a good basis for implementation of oral health programs and highlight the risk factors for oral diseases in adults. Moreover, it will guide policy makers in establishing oral health care measures and planning national oral health policy for adults.

The aim of this study was to assess the oral health status among adult employees in Kuwait.

## Materials and Methods

This cross-sectional study was conducted among adults working at the Ministries Complex and the Housing Authority in Kuwait. The study protocol was approved by the Ethical Research Committee of School Oral Health Program, Kuwait-Forsyth. This study was conducted in accordance with the laws of the State of Kuwait, rules and regulations of the Ministry of Health, and was in full accordance with the World Medical Association Declaration of Helsinki.

A convenience sample of adults (N = 1294) participated in this study. The oral health examinations of the participants were done during morning working hours at the ministries site for about 5 weeks in 2012. The clinical examinations were carried out by 6 trained and calibrated dentists, utilising portable equipment. Informed consent was obtained from the participants before the examinations. Data collection was done on a form designed especially for this study and recorded by the trained recorders.

Oral examinations were performed using a mouth mirror and a WHO ball-tip probe. The inclusion criteria were: adults who gave consent, worked at the site, or were visitors who had government transactions with the ministries. Prior to the initiation of the survey, the examiners and recorders were trained and calibrated by the principal investigator. Examiners and recorders were standardised through a series of training exercises to finalise the diagnostic criteria. During the survey, the principal investigator visited the examination teams on a regular basis to review the diagnostic criteria and examination procedures. After the examinations, all participants were instructed in oral hygiene by two dental hygienists. All participants were given brochures about oral health, toothbrushes and toothpastes. Certificates of appreciation from the Ministry of Health, Kuwait, with a statement of recognition were awarded to the head offices of the Ministries Complex and the Housing Authority, as well as to all members of the survey team for their valuable contribution.

The demographic data of the participants were collected: age, gender, nationality, level of education, place of work, socioeconomic status and marital status. Oral health variables were also recorded: visits to dentist, smoking status and treatment urgency. The medical history was also taken.

Caries was recorded in accordance with the WHO diagnostic criteria^33^ using the tooth-based indices for decayed, missing, and filled teeth (DMFT). Radiographs were not taken. Intra- and inter-examiner reliability of the caries diagnoses as determined with the kappa statistic was 0.80.

Oral hygiene was assessed using the debris index simplified (DI-S), which describes the extent of soft deposits, and is one of the 2 components of the simplified oral hygiene index (OHI-S) developed by Green and Vermillion.^11^ Clinical data on gingival inflammation and gingival recession were also collected. The presence of oral pain at the time of examination was recorded.

### Statistical Analysis

Data were analysed using Epi-Info 3.5.3 (Centers for Disease Control; Atlanta, GA, USA) and SPSS (IBM; Armonk, NY, USA) for Windows 22.0. Frequency distributions for all the variables were generated. Descriptive statistics including means and standard deviations were calculated for the caries indices. Caries experience was estimated in relation to various sociodemographic variables and oral health habits of the participating adults. An independent-samples t-test was used to test the differences in mean caries experience given two groups and by one-way ANOVA given more than two groups. The chi-squared test was used to assess the association of the proportions of caries-free adults by the categorical variables and to test for associations of the background factors with oral hygiene status. Multivariate analysis (logistic regression) was used to assess the risk factors for caries prevalence with various sociodemographic and other factors. The odds ratios (OR) with 95% confidence intervals were calculated. The significance level used was p < 0.05.

## Results

The survey sample comprised 1294 adult employees, of which 846 (65.4%) were Kuwaiti and 448 (34.6%) were of other nationalities. Their ages ranged from 19 to 77 years, the mean age was 36.2 ± 10.5 years, with 67.6% males and 32.4% females. Most of the participants (90.6%) were government employees, while less than ten percent (9.4%) were from the private sector. More than three-fourths (76.9%) were from the Ministries complex and the rest (23.1%) were from the Housing Authority. Seventy percent of the adults were married. Almost two-thirds (65%) of the adults had either a college or university qualification. More than half of adults (54.5%) reported that they had visited a dentist less than one year ago while, 15.2% had visited in the past 12 months, and nearly one-third (32.1%) had had dental visits more than a year ago. More than one-third of the adults (38.2%) had a monthly income between 710 and 1200 Kuwaiti dinars (KWD). Almost one-third of the adults (30.4%) were current smokers. A majority of the participants (80.8%) had no medical condition. Also, most of the adults (86.6%) had no evident dental problem or were in need of non-urgent dental care, while 13.4% required emergency or urgent dental care. Table 1 summarises the demographic characteristics of the participating adults.

**Table 1:** Sociodemographic characteristics and dental health habits of the participating adults (n = 1294)

Variables	n (%)
Age in years (mean ± SD)	36.2 ± 10.5
**Gender**	
Male	875 (67.6)
Female	419 (32.4)
**Nationality**	
Kuwaiti	846 (65.4)
Non-Kuwaiti	448 (34.6)
**Setting**	
Government	1173 (90.6)
Private	121 (9.4)
**Marital status**	
Yes (married)	907 (70.1)
No (single/divorced/widow)	387 (29.9)
**Level of education**	
Intermediate school or less	105 (8.1)
High school	293 (22.6)
Diploma	55 (4.3)
University and above	841 (65.0)
**Visit to the dentist**	
Less than one year ago	705 (54.5)
One year ago	197 (15.2)
More than one year ago	392 (30.3)
Smoking status	
Non-smoker (no)	900 (69.6)
Smoker (yes)	394 (30.4)
**Medical history**	
Healthy	1046 (80.8)
Has medical condition	248 (19.2)

Overall, the mean DMFT in the adults was 10.3 ± 6.7. The DMFT among 35- to 44-year-olds was 10.7 ± 6.9. The lowest mean DMFT was found in the 19- to 24-year age group (7.8 ± 5.2) and the highest in the 65- to 77-year age group (18.9 ± 8.3) (p < 0.001) (Table 2). The mean number of decayed teeth (DT) was 3.0 ± 3.4, missing teeth (MT) 3.4 ± 3.3, and filled teeth (FT) 4.0 ± 4.3. Females had slightly higher caries experience (DMFT) (11.0 ± 5.9) than males (10.1 ± 7.1) (p = 0.021), and Kuwaitis had a higher DMFT (11.1 ± 6.4) than did non-Kuwaitis (8.9 ± 7.1) (p < 0.001) (Table 3).

**Table 2:** The means of caries indices (DT, MT, FT and DMFT) of adults according to the age groups

Age group	n	DT	MT	FT	DMFT
19–24	113	2.7	2.5	2.5	7.8
24–29	317	2.9	2.4	3.4	8.7
30–34	254	3.1	2.9	3.8	9.8
35–39	173	3.1	3.8	4.4	11.3
40–44	149	2.6	3.3	4.1	10.0
45–49	116	3.2	4.1	5.2	12.5
50–54	84	2.8	4.6	4.7	11.8
55–59	53	3.3	6.2	5.8	15.3
60–64	24	4.2	8.3	3.8	16.2
65–77	11	4.6	7.7	6.6	18.9
Total	1294	3.0	3.4	4.0	10.3
Statistical significance p < 0.05		0.416	<0.001	<0.001	<0.001

**Table 3:** The means of caries indices (DT, MT, FT and DMFT) according to gender, nationality, monthly income and level of education

Variables	n	DT	MT	FT	DMFT
**Gender**					
Male	875	3.4	3.3	3.4	10.1
Female	419	2.2	3.6	5.2	11.0
Statistical significance (p < 0.05)		<0.001	0.147	<0.001	0.021
Nationality					
Kuwaiti	846	2.8	3.6	4.8	11.1
Non-Kuwaiti	448	3.4	3.0	2.5	8.9
Statistical significance (p < 0.05)		0.001	0.003	<0.001	<0.001
**Monthly income**					
Less than 300 KWD	194	3.5	2.3	1.3	7.2
300-700 KWD	334	3.4	3.5	3.4	10.3
710-1200 KWD	494	2.6	3.6	4.8	11.0
>1200 KWD	272	2.8	3.7	5.2	11.7
Statistical significance (p < 0.05)		<0.001	<0.001	<0.001	<0.001
**Level of education**					
Intermediate school or less	105	4.2	3.6	2.8	10.6
High school	293	3.3	3.5	3.3	10.1
Diploma	55	3.0	3.5	4.8	11.3
University and above	841	2.7	3.3	4.3	10.4
Statistical significance (p < 0.05)		<0.001	0.826	<0.001	0.671

The mean DMFT varied by the income level. Adults with a monthly income above 1200 KWD had higher DMFT (11.7 ± 6.2) compared to those earning less than 300 KWD (7.2 ± 6.2) (p < 0.001). Adults who had visited the dentist during the last 12 months (11.8 ± 6.6) had a higher DMFT than those who had visited more than one year prior to this study (8.2 ± 6.4) (p < 0.001). The mean DMFT varied by treatment urgency. Adults who had a medical condition had higher DMFT scores (11.7 ± 7.1) than did those who were healthy (10.1 ± 6.6) (p = 0.001). Also, the mean DMFT among married adults was higher (10.8 ± 7.0) compared to those who were single (9.3 ± 6.0) (p < 0.001) (Table 4).

**Table 4:** The means of caries indices (DT, MT, FT and DMFT) according to visit to the dentist, medical history and marital status

Variables	n	DT	MT	FT	DMFT
**Visit to the dentist**					
< one year ago	705	2.9	3.8	5.1	11.8
One year ago	197	3.0	3.0	3.6	9.6
> one year ago	392	3.2	2.8	2.2	8.2
Statistical significance (p < 0.05)		0.483	<0.001	<0.001	<0.001
**Medical history**					
Healthy	1046	3.0	3.2	3.9	10.1
Has medical condition	248	3.0	4.4	4.3	11.7
Statistical significance (p < 0.05)		0.673	<0.001	0.321	0.001
**Marital status**					
Married	907	3.0	3.7	4.1	10.8
Not married	387	3.0	2.6	3.7	9.3
Statistical significance (p < 0.05)		0.854	<0.001	0.108	<0.001

There were statistically significant differences in the mean DT between gender; males had higher DT scores (3.4 ± 3.6) than did females (2.2 ± 2.5) (p < 0.001). Moreover, Kuwaitis had lower DT scores (2.8 ± 3.1) compared to other nationalities (3.4 ± 3.8) (p = 0.001) (Table 3). Adults with an intermediate- or lower educational level had a higher DT (4.2 ± 4.3) compared to those with a university education (2.7 ± 3.2) (p < 0.001). Statistically significant differences were also observed between the mean FT according to gender (males = 3.4 ± 4.2 vs females = 5.2 ± 4.4) (p < 0.001), nationality (Kuwaiti = 4.8 ± 4.5 vs other nationalities 2.5 ± 3.7) (p < 0.001), level of education, monthly income and dental visits.

The proportion of caries-free adults was 28%. A larger percentage of females – more than one-third (34.6%) – were caries-free, compared to one-fourth of males (24.8%) (p < 0.001). Also, higher percentages of adults with a university education or above (30.6%) were caries-free compared to those with intermediate schooling or less (14.3%) (p = 0.004). Almost one-third of non-smokers (30.8%) were caries-free vs 21.6% of smokers (p < 0.001).

The mean number of teeth present (total number of natural teeth) in the participants was 28.6. Almost all of the adults had natural teeth and only 0.5% were edentulous. Less than 10% of the adults (9.9%) had remaining roots. Only 6.1% of adults had root caries. Overall, the proportion denture wearers was only 1%.

In multivariate analysis, adults with poor oral hygiene (OR = 1.5; 95% CI = 1.2-2.1; p = 0.001), those with intermediate schooling or lower (OR = 2.6; 95% CI = 1.4-4.7; p = 0.002), Kuwaitis (OR=1.3; 95% CI=1.0-1.7; p = 0.046), occurrence of oral pain (OR=1.4; 95% CI=1.0-1.8; p = 0.016), and in those requiring emergency or urgent dental care (OR = 4.6; 95% CI = 2.6-8.0; p < 0.0001) were statistically significantly associated with caries risk (Table 5).

**Table 5:** Estimated relative risks (odds ratio) and their 95% confidence intervals for caries prevalence according to various factors

	OR	95% CI	Statistical significance (p < 0.05)
**Oral hygiene**			
Good+Fair	1.0	-	
Poor	1.59	1.20 – 2.12	0.001
**Level of education**			
University and above	1.0		
Intermediate school or less	2.61	1.43 – 4.79	0.002
High school	1.12	0.81 – 1.56	0.475
Diploma	1.25	0.66 – 2.37	0.477
**Nationality**			
Non-Kuwaiti	1.0		
Kuwaiti	1.30	1.00 – 1.70	0.046
**Oral pain**			
No	1.0	-	
Yes	1.40	1.06 – 1.84	0.016
**Treatment urgency**			
No evident problem and non-urgent dental care	1.0	-	
Emergency or urgent dental care	4.61	2.65 – 8.02	0.000

Of the 1294 adults, 19.6% had good oral hygiene, 36.1% fair and 44.4% had poor oral hygiene. More than half (57.5%) of the males had poor oral hygiene, while only 16.9% of the females had poor oral hygiene (p < 0.001) (Table 6). Two-thirds of non-Kuwaitis (66.5%) had poor oral hygiene, compared to one-third (32.6%) of Kuwaitis (p < 0.001). More adults with a diploma qualification had good and fair oral hygiene compared to those with an intermediate school (grades 6 to 9) or lower education (25.5%/43.6% vs 5.7%/21.9%). Fewer participants with a diploma had poor oral hygiene when compared to those with an intermediate-school or lower education (30.9% vs 72.4%) (p < 0.001). The percentage of adults with poor oral hygiene was highest among those who had their dental visit more than one year ago (55.4%) when compared to those, less than a year ago (37.2%) (p < 0.001). Higher percentages of smokers (62.9%) had poor oral hygiene when compared to non-smokers (36.2%), and fewer smokers had good oral hygiene than non-smokers (8.1% vs 24.6%) (p < 0.001). Less than half (45.7%) of adults had gingival recession in at least one tooth. The mean number of teeth with gingival recession was 3.3. Very few adults (2.7%) had a soft tissue abnormality.

**Table 6:** Distribution of the oral hygiene status of the adults according to gender, nationality, level of education, visit to the dentist and smoking status

Variables	n	Oral hygiene status (% of adults)			Statistical significance (p < 0.05)
		Good	Fair	Poor	
**Gender**					
Male	875	10.2	32.3	57.5	
Female	419	39.1	43.9	16.9	<0.001
**Nationality**					
Kuwaiti	846	27.0	40.4	32.6	
Non-Kuwaiti	448	5.6	27.9	66.5	<0.001
**Level of education**					
Intermediate school or less	105	5.7	21.9	72.4	
High school	293	13.7	32.4	53.9	
Diploma	55	25.5	43.6	30.9	
University and above	841	22.9	38.6	38.4	<0.001
**Visit to the dentist**					
< one year ago	705	23.8	39.0	37.2	
One year ago	197	18.3	33.5	48.2	
> one year ago	392	12.5	32.1	55.4	<0.001
**Smoking status**					
Non-smoker	900	24.6	39.2	36.2	
Smoker	394	8.1	28.9	62.9	<0.001

Nearly one-third (32.9%) of adults had perceived oral pain at the time of examination. Higher percentage of other nationalities (41.7%) had oral pain when compared to Kuwaitis (28.3%) (p < 0.001) (Table 7). Nearly half of adults (46.7%) with an intermediate or lesser education had oral pain as compared to less than one-third (29.7%) with a university education (p = 0.002). Oral pain was more prevalent among the adults with poor oral hygiene than among those with good and fair oral hygiene, with 36.3% and 30.3%, respectively (p = 0.014). Oral pain varied by income level and was more prevalent among adults (50.5%) with low income. Also, oral pain was more prevalent among those adults (62.3%) who needed emergency or urgent dental care.

**Table 7:** Occurrence of oral pain in the adults according to nationality, level of education, oral hygiene status, income level and treatment urgency

Variables	n	Yes (%)	No (%)	Statistical significance (p < 0.05)
**Nationality**				
Kuwaiti	846	28.3	71.7	
Non-Kuwaiti	448	41.7	58.3	<0.001
**Level of education**				
Intermediate school or less	105	46.7	53.3	
High school	293	36.9	63.1	
Diploma	55	34.5	65.5	
University and above	841	29.7	70.3	0.002
**Oral hygiene**				
Good+Fair	720	30.3	69.7	
Poor	574	36.2	63.8	0.014
**Monthly income**				
Less than 300 KWD	194	50.5	49.5	
300-700 KWD	334	36.8	63.2	
710-1200 KWD	494	27.3	72.7	
More than 1200 KWD	272	25.7	74.3	<0.001
**Treatment urgency**				
No evident problem and non-urgent dental care	1120	27.1	72.9	
Emergency or urgent dental care	174	62.3	37.8	<0.001


## Discussion

The need for preventive oral health programs in a country is assessed by conducting oral health surveys among different age groups of a population. Most oral health surveys done worldwide have been among children, because of the feasibility and easy access to this age group, e.g. through nurseries, schools, and other locations. This cross-sectional study was conducted to assess the oral health status among adult employees (N = 1294) in Kuwait. In such a population, poor oral health may limit daily activities and lead to work absenteeism due to oral disease. For scoring dental caries, the DMF index was used. This is a well-accepted measure of caries prevalence and may reflect the actual caries experience within the population studied. The debris index simplified (DI-S) of the simplified oral hygiene index (OHI-S) was chosen for this study, as it has been widely used to evaluate the level of oral hygiene in epidemiological studies.

Differences in oral health status reveal not only behavioural characteristics, but also several socioeconomic and environmental factors for a given country and within certain population groups among adults.^27,29,30^ In this study, the mean DMFT of all age groups taken together was 10.3. The mean DMFT among 35- to 44-year-olds was 10.7. In a previous oral health survey in Kuwait among adults ages 19 and ≥65, the mean DMFT was 3.8 and 22.8, respectively.^4^ In the same study,^4^ the mean DMFT was 8.6 among 35- to 44-year-olds, which was lower than in this study. In a Hungarian adult population,^18^ the mean DMFT was higher than in this study for different age groups, from 20- to 24-year-olds (12.8) to 65- to 75-year-olds (21.9). Similarly, the mean DMFT in adult workers in Brazil^3^ among 20- to 64-year-olds was 14.6, which was higher than for the adults in this study.

In comparison with the worldwide caries levels provided by the WHO for 35- to 44-year-olds among different countries,^25,26,27^ in which a mean DMFT of 9.0 to 13.9 for 35- to 44-year-olds is considered moderate, adults in the present study were found to have a moderate dental caries level with 10.7. In an Iranian study among adults,^14^ the mean DMFT was 11.0, slightly higher than this study. In Lebanon, the mean DMFT for adults was 16.0,^9^ also higher than this study. A study done on Turkish adults showed a higher mean DMFT (12.6)^23^ than that found in the present study. Furthermore, a national oral health survey in Turkey among 35- to 44-year-olds showed a mean DMFT (10.8),^10^ similar to that of the present study. In our study, the mean DMFT was lower than in studies among adults in France (14.6).^7,13,31^ The mean DMFT in this study was much lower than in all locations of the International Collaborative Studies (ICS II) survey, in which the DMFT for 35- to 44-year-olds ranged from 11.7 to 20.6 among the countries.^8^ The mean DMFT of the present study was lower than that among adult workers in Brazil (19.6)^3^ and Hungary (15.4)^18^ and was higher than among adults in China (2.1).^32^

The mean DMFT was lowest for the 19- to 24-year age group and highest in the oldest age cohort in this study. Similarly, the mean caries indices increased with age in the previous study in Kuwait.^4^ The increase of mean DMFT with age is expected and agrees with the results of other studies.^25,32^ In the present study, mean DMFT for 65- to 77-year-olds was 18.9, which was lower than the previous study in Kuwait (22.8).^4^ Also, in the present study, it was lower than in International Collaborative Studies (ICS II) survey,^8^ in which the DMFT for 65- to 74-year-olds varied between 23.7 and 28.8. In contrast, among 65- to 74-year-old adults in Germany, a decrease in DMFT was recorded from 1997 (23.6) to 2014 (17.7).^16^ Caries experience was higher (21.9) among 65- to 75-year-old adults in Hungary.^18^ Also, mean DMFT was higher (25.8) in 65- to 74-year-old Turkish adults in a national oral health survey,^10^ and in another study in Turkey it was (22.2) when compared to this study.^23^ In China, the mean DMFT was lower (12.4) among 65- to 74-year-olds.^32^

Kuwaitis had higher DMFT and caries prevalence when compared to non-Kuwaitis in this study. FT (4.0) was the major component of the DMFT index, with a score of 4.8 for the Kuwaitis. The high mean number of filled teeth was probably due to dental services being free of charge and easily accessible in the government sector for Kuwaiti adults. Most of the Kuwaiti adults might have sought restorative treatment due to easy access to oral health services in Kuwait. The mean number of filled teeth was much lower, varying from 0.1 to 1.2, in an earlier study among adults in Kuwait.^4^ In France, the mean number of filled teeth was 10.4 among adults,^13^ but 1.8 among Iranian adults.^14^ Similarly, in Lebanon,^9^ more than one-third (34%) of the high DMFT index was due to filled teeth. High proportions of filled teeth (71%) were noted in France^13^ and in the UK (63%).^24^ A decline in filled teeth has been observed in UK.^6,7^

The overall mean decayed teeth component was 3.0 in this study. A slightly higher mean DT (3.1) was observed in the previous study in Kuwait.^4^ Mean DT was slightly lower among young adults in Iran (2.6).^12^ In this study, decayed teeth were observed more frequently among adults with lower income levels. This was consistent with results from Lebanon.^7^ The overall mean number of missing teeth was 3.4 in this study, whereas it was higher in the previous study in Kuwait (4.6).^4^ In Saudi Arabia^2^ and Iran,^14^ the mean MT was 4.2 and 6.6, respectively, higher than those of this study. In Morocco, more than half (55%) of mean DMFT was due to the MT component.^34^ In another study, tooth loss in Jordanian adults younger than 40 years old was mainly due to caries.^28^

In this study, overall, gender variability was observed in each of the DMF components of the mean caries indices. Males had more untreated decayed teeth but fewer fillings, while females had more filled teeth, showing that females were more likely to have sought restorative treatment than males. Similar gender variation was observed among adults in Lebanon.^9^ The number of filled teeth increased with higher incomes in this study. This finding is consistent with results from Lebanon.^9^ The mean number of filled teeth was greater in those with a higher level of education in this study. The number of FT increased in those adults with a diploma and a university-level education. Similarly, higher FT values were observed among Iranian adults with higher levels of education.^14^

In the present study, less than half (44.4%) of the participants had poor oral hygiene. Higher proportions – almost two-thirds of the participants – had soft deposits and 46% had severe gingivitis in the Kuwait study published in 2002.^4^

Overall, the proportion of adults wearing dentures was only 1% in this study. Compared to this study, higher percentages of adults (12.1%) were using dentures in an earlier study in Kuwait,^4^ whereas 3.2% of adults in Lebanon wore dentures.^9^ In this study, only 0.5% of the participants were edentulous. The prevalence of edentulousness was higher (2.6%) in Turkish adults.^10^ A higher percentages of adults (3.2%) in Lebanon were also edentulous.^9^

In this study, more than half of adults (55%) reported that they had visited a dentist during the last 12 months. In contrast, only about one-third had visited a dentist during the previous 12 months in the earlier Kuwait study.^4^ The percentage of middle-aged adults who had visited the dentist during the past 12 months was also low internationally.^8^

In the present study, one-third (33%) of the participants had perceived oral pain at the time of examination. In previous studies in Kuwait, toothache (69%) was as the main reason for dental visits among adults^4^ and (70%) among University students.^1^ About 40% of pregnant women in Kuwait had experienced dental pain in the previous 6 months and half them had visited a dentist for dental pain.^15^

Several strengths of this study were that calibrated and well-trained dentists performed the examinations according to WHO criteria, which were also followed for recording the data. Furthermore, this comprehensive survey, which was conducted among adults in Kuwait, is considered the first after the Gulf war in 1990. The limitations of this study were that it was performed on a convenience sample of adult employees at two sites – the Ministry Complex and Housing Authority only – and thus may not be representative of all adults in Kuwait. Moreover, this study was based on a cross-sectional survey in only a selected group of adults; hence, it is difficult to discuss the various factors that may influence the oral health status of Kuwaiti adults in general. Future studies can be done with a wider range of the adult population in Kuwait.

Results from the survey will aid in the formulation of strategies to meet the oral health needs among the adults and to give a good basis for implementation of oral health programs for adults.

## Conclusion

Oral hygiene measures and oral health education need to be reinforced among adults in Kuwait. Implementing oral health programs is necessary, and emphasis should be placed on introducing oral health promotion activities in workplaces to foster oral hygiene practices among adults in Kuwait.

## Acknowledgements

We express our appreciation to all the adult employees who participated in the survey and also to the Ministry authorities for their wholehearted support. We gratefully acknowledge the support of all the survey team members, staff and coordinators: Dr. Sabiha Al-Mutawa, Dr. Eman Al-Menezaa, Dr. Ghosun Al Ali, Dr. Fotooh Al Ali, Dr. Shaheen Al Hawaj, Dr. Anood Al Rajhy, Dr. Meshari Al Nafisi, Dr. Turky AlMujaweb, Dr. Mary Yunan, the dental hygienists Rabaa Al Gareeb, Sheyma Al Alaqil, and the dental assistants Laila Khajah and Rabab Khajah of the oral health survey team, School Oral Health Program, Kuwait, for their cooperation and assistance.
